# 

**Published:** 1964-04

**Authors:** 


BRISTOL MEDICO-CHIRURGICAL JOURNAL

*(Incorporating the Medical Journal of the South-West)*

Established 1883

VOL. 79 (ii) April 1964 NO. 292

### Contents

*PUBLISHED QUARTERLY*

*ANNUAL SUBSCRIPTION* 16/- *POST FREE*

PUBLISHED UNDER THE AUSPICES OF THE
BRISTOL MEDICO-CHIRURGICAL SOCIETY

### *Editorial Committee*

Dr. N. J. Brown, Southmead Hospital, Bristol. *Joint Editor.*

Dr. A. B. Raper, Department of Pathology, Bristol Royal Infirmary. *Joint Editor.*

Dr. J. Macrae, Ham Green Hospital.

Dr. R. C. Wofinden, Central Clinic, Bristol.

Mr. A. E. S. Roberts, Medical Library, University of Bristol. *Secretary.*

### *Local Correspondents*

*Bath* . . Mr. A. D. Bateman, 3 The Circus, Bath.

*Exeter* . . Dr. L. N. Jackson, Mount Jocelyn, Crediton, Devon.

*Gloucester* . Dr. R. F. Jarrett, 9 College Green, Gloucester.

*Plymouth* . Dr. C. R. Croft, Lexden, Hartley Av., Plymouth.

*Taunton* . Dr. M. McAlley, 1 The Crescent, Taunton.

*Torquay* . Dr. A. Robinson Thomas, 20 Devon Square, Newton Abbot, Devon.

*Truro* . . Dr. F. D. M. Hocking, St. Andrews, Perranporth, Cornwall.

*Yeovil* . . Mr. J. A. Vere Nicoll, Knapp House, Yeovil, Somerset.

### NOTICE TO CONTRIBUTORS

Original articles are invited, provided they have not been published elsewhere. Notes of forthcoming events, meetings held, degrees conferred, staff appointments, and other local news are welcomed. The editorial committee reserves the right to make literary corrections.

Illustrations should always be supplied ready for reproduction. All re-touchings or other operations required will be charged to the author. Line drawings should be drawn in Indian ink. We regret that we must ask authors to pay for making blocks, if this is necessary.

J. W. ARROWSMITH LTD., WINTERSTOKE ROAD,
BRISTOL.

### Notes and News

Mr. Robert V. Cooke becomes President of the Moynihan Club for 1964–65, and of the Section of Proctology of the Royal Society of Medicine.

Mr. J. Angell James has been elected Vice-President of the British Association of Otolaryngologists for 1963–64.

### Obituary

H. Lovell Hoffman

W. A. Jackman

E. Watson-Williams

R. J. Irving-Bell

### Examination Results

M.R.C.P. M. S. Knapp
University of Bristol, M.D., B. O. Amure
Ph.D., J. S. Cooper

### Appointments

SOUTH WESTERN REGIONAL HOSPITAL BOARD
CONSULTANTS, SPECIALISTS AND REGISTRARS APPOINTED DURING THE PERIOD JULY, 1963 TO DECEMBER 1963

| *Name* | *Appointment* |
| --- | --- |
| | *BRISTOL* |
| Keen, G., M.S., F.R.C.S. | Consultant Thoracic and Intra-Cardiac Surgeon, Bristol Clinical Area. *Formerly:* Senior Registrar in Thoracic Surgery, The Westminster Hospital, London. |
| Ratliff, A. H. C., F.R.C.S. | Consultant Orthopaedic Surgeon, Bristol Clinical Area. *Formerly:* Consultant Orthopaedic Surgeon, North West Metropolitan Regional Hospital Board. |
| Marmion, V. J., M.R.C.P. (Edin.), F.R.C.S. (Edin.) | Consultant Ophthalmologist, Bristol Clinical Area. *Formerly:* Senior Registrar, St. Paul's Eye Hospital, Liverpool. |
| Heath, I. D., M.B., CH.B. (Edin.) | Medical Registrar, Cossham-Frenchay Hospital Group. *Formerly:* S.H.O. in General Medicine, General Hospital, Nottingham. |
| Banerjee, R. K., M.B.B.S. (Calcutta) | Registrar in Diseases of the Chest, Ham Green Hospital/Bristol Chest Clinic. *Formerly:* Senior Resident House Officer, The Grange Hospital. |
| Bishara, Z., M.B., CH.B., F.R.C.S., M.R.C.O.G. | Registrar in Obst. & Gynae., Southmead/Frenchay Hospitals. *Formerly:* Locum Registrar in Obst. & Gynae. The Radcliffe Infirmary. |

Calvert, D. G., M.B., B.S., F.R.C.S. — Senior Surgical Registrar, Southmead Hospital/United Bristol Hospitals.
*Formerly:* Locum Senior Surgical Registrar, Bristol Royal Infirmary.

Day, K. A., M.B., CH.B. — Registrar in Psychiatry, Glenside and Barrow Hospital Group.

Harley, N. F., M.B., B.S., D.A. — Anaesthetic Registrar, Southmead/Frenchay Hospitals.
*Formerly:* General Practice.

Fry, D. I., M.A., B.M., B.CH., D.A. — Anaesthetic Registrar, Southmead Hospital.
*Formerly:* Anaesthetic Registrar, St. Luke's Hospital.

Mahoney, M. P., M.B., B.CH. — Medical Registrar, Southmead Hospital.
*Formerly:* Medical Registrar, Manchester Royal Infirmary.

### *BATH*

John, H. T., M.S., F.R.C.S. (Eng.) — Consultant Surgeon, Bath Clinical Area.
*Formerly:* Lecturer in Surgery, University of Bristol.

Bourke, M. T., M.B., CH.B., D.C.H. — Clinical Assistant in Paediatrics, Royal United Hospital.
*Formerly:* General Practice.

Bazeley, R. W., M.B., B.S. — Clinical Assistant in General Medicine, Bath Group of Hospitals.
*Formerly:* General Practice, and Cl. Asst. in General Medicine, Bath.

Burrowes, W. L., M.D., M.R.C.P. (Ireland) — Clinical Assistant in General Medicine, Bath Group of Hospitals.
*Formerly:* General Practice, and Cl. Asst. in General Medicine, Bath.

Ellison, J., M.B., CH.B., M.R.C.P. (Edin.) — Clinical Assistant in General Medicine, Bath Group of Hospitals.
*Formerly:* General Practice.

Ronn, H. H., M.B., B.S., M.R.C.P. (Lond.) — Clinical Assistant in General Medicine, Bath Group of Hospitals.
*Formerly:* General Practice, and Clinical Asst. in General Medicine, Bath.

Aihie, E., M.A., B.M., B.CH. — Registrar in Psychiatry, Mendip Hospital, Wells.
*Formerly:* Locum J.H.M.O. in Psychiatry, Mendip Hospital.

Desai, S. K. H., M.B., B.S. — Registrar in Anaesthetics, Bath Group of Hospitals.
*Formerly:* S.H.O. in Anaesthetics, St. Martin's Hospital, Bath.

Newth, J. B., B.A., M.B., B.CHIR. — Medical Registrar, Bath Group of Hospitals.
*Formerly:* Locum Medical Registrar, Bath Group of Hospitals.

Saxena, S., M.B., B.S., D.A. — Registrar in Anaesthetics, Bath Group of Hospitals.
*Formerly:* S.H.O. in Anaesthetics, Royal United Hospital, Bath.

Sil, K. C., M.B., B.S., F.R.C.S. (Glasgow) — Registrar in E.N.T. Surgery, Bath Group of Hospitals.
*Formerly:* Locum E.N.T. Registrar, Enfield Group of Hospitals.

### *SOUTH SOMERSET*

Robinson, D. C., B.M., B.CH., M.R.C.P. (Lond.) — Paediatric Registrar, Musgrove Park Hospital, Taunton/The London Hospital (joint appointment).
*Formerly:* Paediatric Registrar, The London Hospital.

### *DEVON AND EXETER*

Dawson, A. M., M.R.C.S., L.R.C.P., M.R.C.O.G.
Consultant Obstetrician and Gynaecologist, Devon and Exeter Clinical Area.
*Formerly:* S.H.M.O. in Obstetrics, and Gynaecology, The Lewis and Harris Hospitals.

Burgess, W., M.B., B.S., D.C.H., M.R.C.P.
Medical Registrar, North Devon Infirmary.
*Formerly:* Part-time Asst. County Medical Officer, Devon County Council.

Campbell, G. D., M.B., CH.B., F.R.C.S.
Surgical Registrar, Royal Devon and Exeter Hospital.
*Formerly:* Surgical Registrar, Royal Hospital, Sheffield.

DuPreez, H. M., M.B., CH.B., F.R.C.S. (Eng.)
Surgical Registrar, Torbay Hospital.
*Formerly:* Locum Senior Surgical Registrar, Southampton General Hospital.

Espiner, H. J., M.B., CH.B., F.R.C.S., CH.M.
Senior Surgical Registrar, Royal Devon and Exeter Hospitals/United Bristol Hospitals (joint appointment).
*Formerly:* Clinical Research Asst., Bristol Royal Infirmary.

Henderson, K., M.B., CH.B.
Anaesthetic Registrar, Torbay Hospital.
*Formerly:* S.H.O. in Anaesthetics, Torbay Hospital.

Kellock, M. G., M.B., CH.B., D.A.
Anaesthetic Registrar, Royal Devon and Exeter Hospitals.
*Formerly:* S.H.O. in Anaesthetics, Clatterbridge Hospital, Cheshire.

Mathieson, A., M.B., CH.B., D.OBST.R.C.O.G.
G.P. Anaesthetist, Totnes and Brixham Hospitals.
*Formerly:* Locum tenens Anaesthetist, Torbay and Royal Devon and Exeter Hospitals.

Shaw, N. M., M.B., CH.B.
Orthopaedic Registrar, Torbay Hospital/Princess Elizabeth Orthopaedic Hospital.
*Formerly:* Surgical Registrar, Wolverhampton and New Cross Hospitals.

### *PLYMOUTH*

Domeiri, A., M.B., B.CH., F.R.C.S. (Edin.)
Orthopaedic Registrar, Mount Gold Orth. Hospital.
*Formerly:* S.H.O. in General Surgery, Rossendale Hospital.

Forbes, W. R., M.B., CH.B., M.R.C.P. (Edin.), D.C.H., D.OBST.R.C.O.G.
Paediatric Registrar, Plymouth General Hospital/United Bristol Hospitals (joint appointment).
*Formerly:* Paediatric Registrar, South Shields Group of Hospitals.

Knapton, P. J., M.B., CH.B., M.R.C.P. (Lond.)
Senior Medical Registrar, Plymouth General Hospital/United Bristol Hospitals (joint appointment).
*Formerly:* Medical Registrar, Queen Elizabeth Hospital, Birmingham.

Murphy, C. D., M.B., CH.B., F.R.C.S. (Eng.)
Surgical Registrar, Plymouth General Hospital.
*Formerly:* Locum Surgical Registrar, Cheltenham General Hospital.

Pullen, B. W., M.B., B.S.
Registrar in General Medicine and Infectious Diseases, Plymouth General Hospital/The Scott Hospital.
*Formerly:* S.H.O., Falmouth and District Hospital.

### WEST CORNWALL

| | |
|---|---|
| Collins, C. J., M.B., B.CH., B.A.O., D.P.M. | Assistant Psychiatrist (S.H.M.O. grade), St. Lawrence's Hospital, Bodmin. *Formerly:* J.H.M.O. in Psychiatry, Moorhaven Hospital. |
| Hilal, R., M.B., B.S., M.R.C.P. (Glasgow) | Medical Registrar, Camborne-Redruth/ Tehidy Hospitals. *Formerly:* S.H.O. Tehidy Hospital. |

### NORTH GLOUCESTERSHIRE

| | |
|---|---|
| Colman, R. S., M.B., B.S. | Surgical Registrar, Cheltenham General Hospital. *Formerly:* Locum, St. Albans City Hospital, Herts. |
| Ellwood, R., M.B., B.S., M.R.C.O.G. | Registrar in Obstetrics and Gynaecology, Gloucestershire Royal Hospital. *Formerly:* Locum Registrar and S.H.O., Southend-on-Sea General Hospital. |
| Adams, J. F. R., M.B., B.S. | Clinical Assistant in Geriatrics, Delancey Hospital. *Formerly:* General Practice. |
| Clyde, J., M.B., B.S., F.R.C.S. (Edin.) | Surgical Registrar Gloucestershire Royal Infirmary. *Formerly:* Locum Registrar, Lewisham Hospital. |

### UNITED BRISTOL HOSPITALS

July to December 1963

| *Name* | *Appointment* |
|---|---|
| Fletcher, J. P., F.D.S.R.C.S. | Hon. Consultant in Dental Medicine. |
| John, H. T., D.F.C., F.R.C.S. | Hon. Consultant in Surgery. |
| Hunt, A. C., M.D., B.S. | Hon. Consultant in Pathology. |
| Brooks, A. W., L.D.S.R.C.S. | S.H.D.O., Dental Surgery. |
| Celestin, L. R., F.R.C.S. | Senior Registrar in Surgery. |
| Davidson, C. M., F.R.C.S.E. | Senior Registrar in Surgery. |
| Mills, P. V., F.R.C.S. | Senior Registrar in Ophthalmology. |
| Knapp, M. S., M.B., CH.B., M.R.C.P. | Registrar in Medicine. |
| Williams, E. R., M.B., CH.B. | Registrar in Medicine. |
| Pantazopoulos, T., M.D. | Registrar in Orthopaedics. |
| Faulkner, D., F.F.A.R.C.S. | Registrar in Anaesthetics. |
| Crooke, J. W., M.B., B.S., D.A. | Registrar in Anaesthetics. |
| Voyce, M. A., M.B., CH.B., D.C.H. | Registrar in Paediatrics. |
| Pottinger, R. F., M.B., CH.B., D.O. | Registrar in Ophthalmology. |
| Duke, P. S., M.B., B.S., D.O. | Registrar in Ophthalmology. |
| Hart, C. T., M.B., B.S. | Registrar in Ophthalmology. |
| Mehta, F. F., L.D.S.R.C.S. | Registrar in Dental Surgery. |
| Halazonetis, J., L.D.S.R.C.S. | Registrar in Dental Surgery. |
| Hilton-Bailey, I., B.D.S. | Registrar in Dental Surgery. |
| Miles, P., M.R.A.C.P., | Registrar in Cardiology. |
| Dovey, P., M.B., CH.B. | Registrar in Radiology (Trainee). |
| Payne, R., M.B., CH.B., D.OBST., R.C.O.G. | Registrar in Radiology (Trainee). |
| Steel, Miss Y., M.B., CH.B. | Registrar in Radiology (Trainee). |

Printed by

J. W. Arrowsmith Ltd., Bristol

